# Public assistance program and food diversity among older people: a cross-sectional study using the Japan Gerontological Evaluation Study data: Public assistance program and food diversity

**DOI:** 10.1186/s12939-025-02494-3

**Published:** 2025-05-12

**Authors:** Kotone Tanaka, Daisuke Nishioka, Atsushi Nakagomi, Keiko Ueno, Kazushige Ide, Shiho Kino, Nobuko Murayama, Katsunori Kondo

**Affiliations:** 1 School of Nutrition and Dietetics, Faculty of Health and Social Services, Kanagawa University of Human Services, 1-10-1 Heiseicho, Yokosuka, Kanagawa 238-8522 Japan; 2 Department of Social Impact Assessment and Evaluation, Graduate School of Medicine, School of Public Health, Kyoto University, Floor 2, Science Frontier Laboratory, Yoshidakonoe- cho, Sakyo-ku, Kyoto-shi, Kyoto, 606-8315 Japan; 3 Department of Social Preventive Medical Sciences, Center for Preventive Medical Sciences, Chiba University, 1-33 Yayoicho, Inage-ku, Chiba, Chiba 263-8522 Japan; 4 Department of Social Epidemiology, Graduate School of Medicine and School of Public Health, Kyoto University, Floor 2, Science Frontier Laboratory, Yoshidakonoe-cho, Sakyo-ku, Kyoto-shi, Kyoto, 606-8315 Japan; 5 Department of Community Building for Well-being, Center for Preventive Medical Sciences, Chiba University, 1-33 Yayoicho, Inage-ku, Chiba, Chiba 263-8522 Japan; 6 Department of Preventive Oral Health Care Sciences, Graduate School of Medical and Dental Sciences, Institute of Science Tokyo, 1-5-45 Yushima, Bunkyo-ku, Tokyo, Japan; 7 Department of Health and Nutrition, Faculty of Human Life Studies, University of Niigata Prefecture, Ebise-471, Niigata, Japan; 8 Research Department, Institute for Health Economics and Policy, Association for Health Economics Research and Social Insurance and Welfare, Tokyo, 105-0003 Japan

**Keywords:** Dietary inequities, Food diversity, Japan, Poverty, Public assistance

## Abstract

**Background:**

Food diversity plays an important role in people’s healthy and affluent lives. However, poverty and eating alone can create multi-dimensional barriers to food diversity. Although public assistance programs guarantee a minimum income to people in need, financial support alone may not be sufficient to support the health of people in poverty. This study aimed to identify the differences in food diversity intake between older recipients of public assistance and non-recipients.

**Methods:**

This cross-sectional study utilized data from the Japanese Gerontological Evaluation Study (2022), involving 14,467 participants aged 65 years and older. The Dietary Variety Score (DVS), ranging from 0 to 10 (higher scores indicate higher dietary variety), assessed dietary diversity based on the regular consumption of ten food groups. Receiving public assistance was categorized as “yes” or “no.” Eating together was defined as eating with others every day. To assess the relationship between receiving public assistance and the DVS, we calculated the unstandardized coefficient (β) and *p* values using a general linear model. Additionally, the interaction between public assistance and eating together was examined. As covariates, we adjusted for sociodemographic factors such as age, disease, marital status, and living alone.

**Results:**

Men recipients of public assistance had a lower DVS, even after adjusting for sociodemographic factors (adjusted β: -0.72, *p* = 0.04). For women, no association was seen between receiving public assistance and a lower DVS (adjusted β: -0.19, *p* = 0.66). An interaction between public assistance and eating together was observed among men (*p* = 0.07).

**Conclusions:**

Even after adjusting for sociodemographic factors, men recipients of public assistance have less food diversity than non-recipient men. Men recipients were more likely to increase their food diversity by eating together. To ensure recipients’ rights to food security, the public assistance program should provide additional support to integrate recipients into communities that enable them to eat together in addition to providing financial support.

**Supplementary Information:**

The online version contains supplementary material available at 10.1186/s12939-025-02494-3.

## Background

Sufficient and healthy food should be universally guaranteed to all people. Food security is an important factor that has been directly linked to unhealthy lifestyles and poor health [1–4]. The Food and Agriculture Organization of the United Nations [5] has made a proposal to ensure food security worldwide, defining it as “when all people, at all times, have physical and economic access to sufficient, safe and nutritious food that meets their dietary needs and food preferences for an active and healthy life.” Food diversity can be considered one of the key indicators for evaluating the quality of food security that meets people’s dietary needs and food preferences. Indeed, the enrichment of food diversity may lead to adequate nutrient intake [6, 7], thereby improving the health [8–11] and quality of life of people [12–14].

However, socio-economic factors can be a barrier to sufficient food diversity. For example, Chalermsri et al. reported that a higher wealth index had a positive association with food diversity among older Thai participants [15]. This phenomenon was also seen in the Japanese context. Fukuda found that a lower income had a negative association with food diversity among older participants [16]. A possible explanation for the association between an individual’s low socioeconomic status and lower food diversity is that people with better economic status can spend more money on food [6, 17].

To reduce dietary disparities stemming from income inequalities in Japan, the public assistance program (*seikatsu-hogo* in Japanese) guarantees a minimum healthy and cultural standard of living for people living in poverty [18]. The public assistance program is a welfare system for individuals from low-income backgrounds who require financial support. It provides eligible households with monthly income benefits to meet the minimum standard of living. In December 2022, 1.6% of the Japanese population received public assistance, and 55.3% of the recipients were older adults [19]. Older recipients are no longer employed; therefore, health maintenance is an important mission for them. Consequently, public assistance programs should contribute to ensuring adequate food diversity for such recipients.

Moreover, impoverished individuals, such as recipients of public assistance, not only face financial problems but also multidimensional and complex problems in maintaining food diversity [20]. For example, many recipients live alone [19], have depression [21], and are often socially isolated [22], which are known factors that reduce food diversity [15, 23–26]. In addition, poverty has been found to reduce cognitive restraint in eating behavior [27, 28], because poverty itself reduces cognitive capacity [29], which may contribute to an unhealthy diet. Therefore, poverty may have a multidimensional impact on the reduction in food diversity through non-economic pathways, and financial support can only address one aspect of poverty.

Social connectedness may be effective in mitigating multidimensional dietary disadvantages. For example, eating together has been suggested as a potential intervention for improving dietary diversity [25, 30, 31]. In addition, preparing meals, including food shopping assistance and receiving food [16], which are likely to occur simultaneously with eating together, are associated with improved dietary diversity due to a reduction in unhealthy food choices [32] and fewer missed meals [30]. Furthermore, eating together reduces depression [25, 26] and enhances happiness [33]. Therefore, recipients of public assistance are more likely to improve their food diversity by eating together. However, no study has examined this hypothesis.

Therefore, the present study aimed to investigate the differences in food diversity intake between recipients of public assistance and the general population. In addition, we examined the interaction effects of eating together as a potential factor that could improve food diversity intake among recipients.

## Methods

### Study design and participants

We used cross-sectional data from the Japan Gerontological Evaluation Study (JAGES), an ongoing cohort study exploring the social, environmental, and behavioral factors related to the loss of health, especially functional decline or cognitive impairment, in individuals aged 65 years and older [34]. Between November and December 2022, self-reported questionnaires on food diversity were mailed to 38,676 participants aged 65 years and older, and 25,700 participants completed and returned the questionnaire (response rate: 66.4%). We excluded participants based on the following criteria: (1) those who did not provide informed consent or information on gender, age, or residence area (*n* = 1,863); (2) those who answered “applying for public assistance” or “received public assistance in the past” to the question about receiving public assistance (*n* = 89); (3) and those with missing values on the variables used in the present study (*n* = 9,281). The final analysis included 14,467 participants. All participants were informed that participation in the study was voluntary and that completing the questionnaire, selecting the acceptance checkbox, and returning it via mail indicated their consent to participate in the present study.

### Exposure

#### Public assistance status

Participants were asked, “Do you currently receive public assistance?” There were four response options: “not receiving public assistance,” “receiving public assistance,” “applying for public assistance,” and “received public assistance in the past.” Those who responded “not receiving public assistance” (non-recipients) and “receiving public assistance” (recipients) were included in the present study.

#### Eating together

Eating together was assessed by asking, “How often do you eat meals with someone else?” The possible answers were categorized into two groups: eating together (every day) or not eating together (few times a week, few times a month, few times a year, or rarely).

### Outcome

#### Food diversity

Food diversity was assessed using the Dietary Variety Score (DVS) [35]. The DVS assesses the frequency of intake across 10 food groups pivotal to Japanese cuisine: fish or seafood, meat, eggs, milk, soy products, green/yellow vegetables, fruits, seaweed, potatoes, and fats or oils. The assessment did not include the portion size of intake for each group. These 10 food groups constitute many of the main and side dishes of Japanese cuisine. The frequency options were: (a) daily or almost daily (≥ 5 days a week), (b) once every 2 days, (c) once or twice a week, and (d) hardly ever. Option (a) was assigned 1 point, whereas options (b) to (d) were assigned 0 points each, and the DVS was calculated as the sum of these points. The total score ranges from 0 to 10, with higher scores indicating a higher food diversity. Due to their similar nutrient characteristics, the frequency of dairy products, rather than just milk, was investigated in the present study.

### Covariates

Information was obtained on gender, age, eating together, living arrangement, marital status, education, household income, current medical treatment for diseases, oral frailty risk, Instrumental Activities of Daily Living (IADL), alcohol intake, smoking status, number of remaining teeth, and use of dentures or bridges.

Gender was categorized as men or women. Age was considered a continuous variable. Living arrangements were categorized into two groups: living with someone (spouse, child, or others) or living alone. Marital status was also categorized into two groups: married or unmarried (single, widowed, divorced, or other). Education was categorized into four groups by years of schooling: <10 years, 10–12 years, ≥ 13 years, and others. Household income, expressed per year and including subsidies from public assistance and pensions, was categorized into three groups: <2 million yen, 2–4 million yen, and ≥ 4 million yen. Current medical treatment status includes the presence or absence of each of the following conditions that may affect dietary intake: hypertension, diabetes mellitus, hyperlipidemia, cancer, and depression. Oral frailty risk [36] was categorized into two groups: “at risk” if two or more of the following questions were applicable — “Do you have more difficulty eating hard foods than 6 months ago?” “Do you sometimes choke while drinking tea or soup?” “Do you feel thirsty or have dryness in your mouth?” — otherwise, it was classified as “not at risk.” IADL, assessed using the Tokyo Metropolitan Institute of Gerontology Index of Competence [37], was analyzed as a continuous variable (0–13 points). Alcohol intake was categorized into three groups: current, quit, and never. Similarly, smoking status was categorized into three groups: current, quit, and never. Number of remaining teeth was categorized into five groups [38]: no teeth, 1–4, 5–9, 10–19, and ≥ 20. The use of a denture or bridge was assessed by asking, “Do you use a denture or bridge?” The possible answers were categorized into two groups: yes and no.

### Statistical analysis

We performed all analyses separately for men and women to account for potential differences in the background and diet. First, we used descriptive statistics to compare the characteristics of public assistance recipients and non-recipients. Second, we used a general linear model (GLM) to estimate the unstandardized coefficient (β) and 95% confidence intervals (CI) of the DVS for each category of recipients of public assistance, eating together, and their interaction. To account for the confounding variables, we adjusted for age, living arrangement, marital status, education, household income, current medical treatment of diseases, oral frailty risk, IADL, alcohol intake, smoking status, number of remaining teeth, and use of denture or bridge. It has been shown that public assistance recipients have a lower response rate to social surveys, a higher proportion of misclassification, and missing values for the question regarding the receipt of public assistance than the general population [39]; therefore, we assumed that the missing data observed in the present study did not occur at random. Accordingly, in the present study, we applied complete-case analyses, although there was potential selection bias limiting the participants who were capable of responding to all the JAGES questionnaires, we applied complete-case analyses.

For sensitivity analysis, we used propensity score matching to match the conditions between recipients of public assistance and non-recipients to elucidate the impact of receiving public assistance on food diversity among populations with the same standard of living that would meet public assistance eligibility. To calculate the propensity scores to balance the matching groups, we selected three variables that are evaluated when considering applications for public assistance in Japan: household income, number of household members, and current medical treatment for diseases, as done in a previous study [21]. We used a one-to-one caliper (0.2) matching without replacement. Statistical significance was set at *p* < 0.05. All statistical analyses were performed using R version 4.2.

## Results

Table 1 shows the characteristics of the study participants. The percentage of valid participants was 56.3%. The percentage of missing values ranged from 2.0% (for education) to 13.9% (for household income), and 4.3% of participants did not report if they were recipients of public assistance. More missing values were found among non-recipients than recipients. Among the 14,467 participants who answered all the variables used in the analyses, 123 (0.9%) received public assistance. The mean (standard deviation) age of the participants was 74.3 (6.2) years, and 50.8% were men. For both men and women, recipients of public assistance had a lower mean DVS than non-recipients (1.9 vs. 3.5 for men; 4.1 vs. 4.5 for women). The mean DVS was higher in women than in men. In addition, for both men and women, recipients had fewer instances of “eating together” than non-recipients (18.2% vs. 71.3% for men; 45.6% vs. 69.3% for women). In addition, the proportion of participants with a household income of < 2 million yen was more than four times higher among men recipients than among non-recipients (78.8% vs. 17.3%) and more than twice as high as that among women recipients (70.2% vs. 28.0%). The proportion of participants living alone was more than seven times higher among men recipients than among non-recipients (71.2% vs. 10.0%) and more than twice as high as that among women recipients (49.1% vs. 18.2%).

**Table 1:** Characteristics of the study participants categorized by public assistance

	Men		Women	
	Non-recipients of public assistance	Recipients of public assistance	Non-recipients of public assistance	Recipients of public assistance
n	7283	66	7061	57
Dietary variety score (SD)	3.5 (2.6)	1.9 (2.7)	4.5 (2.6)	4.1 (2.6)
Age, mean (SD)	74.4 (6.2)	74.0 (6.4)	74.2 (6.2)	74.5 (6.4)
Eating together = Everyday, n (%)	5193 (71.3)	12 (18.2)	4892 (69.3)	26 (45.6)
Living status = living alone, n (%)	727 (10.0)	47 (71.2)	1287 (18.2)	28 (49.1)
Number of household members, n (SD)	2.6 (1.3)	1.6 (1.4)	2.4 (1.3)	1.8 (1.0)
Marital status = married, n (%)	1076 (14.8)	50 (75.8)	2427 (34.4)	34 (59.6)
Education, n (%)				
< 10 years	1210 (16.6)	25 (37.9)	1367 (19.4)	12 (21.1)
10–12 years	2980 (40.9)	21 (31.8)	3359 (47.6)	33 (57.9)
≥ 13 years	3042 (41.8)	18 (27.3)	2286 (32.4)	12 (21.1)
Other years	51 (0.7)	2 (3.0)	49 (0.7)	0 (0.0)
Household income, n (%)				
< 2 million yen	1258 (17.3)	52 (78.8)	1979 (28.0)	40 (70.2)
2–4 million yen	3323 (45.6)	7 (10.6)	2797 (39.6)	8 (14.0)
≥ 4 million yen	2702 (37.1)	7 (10.6)	2285 (32.4)	9 (15.8)
Current medical treatment status, n (%)				
Hypertension	3574 (49.1)	27 (40.9)	3085 (43.7)	33 (57.9)
Diabetes mellitus	1322 (18.2)	13 (19.7)	726 (10.3)	12 (21.1)
Hyperlipidemia	1034 (14.2)	6 (9.1)	1497 (21.2)	12 (21.1)
Canser	376 (5.2)	3 (4.5)	253 (3.6)	0 (0.0)
Depression	45 (0.6)	1 (1.5)	68 (1.0)	3 (5.3)
Oral frailty risk (at risk), n (%)	1427 (19.6)	38 (57.6)	1354 (19.2)	14 (24.6)
IADL score, mean (SD)	9.9 (2.3)	9.6 (2.1)	11.3 (2.0)	10.7 (2.2)
Alcohol intake, n (%)				
Current	4442 (61.0)	32 (48.5)	1737 (24.6)	18 (31.6)
Quit	1241 (17.0)	13 (19.7)	589 (8.3)	6 (10.5)
Never	1600 (22.0)	21 (31.8)	4735 (67.1)	33 (57.9)
Smoking status, n (%)				
Current	1141 (15.7)	21 (31.8)	293 (4.1)	6 (10.5)
Quit	4216 (57.9)	28 (42.4)	547 (7.7)	9 (15.8)
Never	1926 (26.4)	17 (25.8)	6221 (88.1)	42 (73.7)
Number of remaining teeth, n (%)				
No teeth	527 (7.2)	10 (15.2)	366 (5.2)	5 (8.8)
1–4	435 (6.0)	12 (18.2)	246 (3.5)	3 (5.3)
5–9	580 (8.0)	16 (24.2)	450 (6.4)	7 (12.3)
10–19	1355 (18.6)	19 (28.8)	1286 (18.2)	10 (17.5)
≥20	4386 (60.2)	9 (13.6)	4713 (66.7)	32 (56.1)
Using denture or bridge = yes, n (%)	4611 (63.3)	44 (66.7)	4397 (62.3)	40 (70.2)
Frequency of intake of food group = daily or almost daily, n (%)				
Fish/seafood	2202 (30.2)	12 (18.2)	2581 (36.6)	20 (35.1)
Meat	2067 (28.4)	14 (21.2)	2865 (40.6)	21 (36.8)
Eggs	3228 (44.3)	23 (34.8)	3585 (50.8)	27 (47.4)
Dairy products	4176 (57.3)	12 (18.2)	4965 (70.3)	39 (68.4)
Soy products	2862 (39.3)	17 (25.8)	3633 (51.5)	24 (42.1)
Green/yellow vegetables	4088 (56.1)	15 (22.7)	5090 (72.1)	39 (68.4)
Fruits	3567 (49.0)	12 (18.2)	4771 (67.6)	34 (59.6)
Seaweed	1478 (20.3)	9 (13.6)	1841 (26.1)	11 (19.3)
Potatoes	871 (12.0)	7 (10.6)	1328 (18.8)	8 (14.0)
Fats/oils	1062 (14.6)	5 (7.6)	1401 (19.8)	8 (14.0)

Abbreviations: IADL, Instrumental Activities of Daily Living; SD, standard deviation

Table 2 shows the results of the GLM used to examine the relationship among DVS, receiving public assistance, and eating together. Among men, receiving public assistance was associated with a lower DVS in the crude model and fully-adjusted model (β: -1.61 [95% CI: -2.23, -0.98] *p* < 0.001; adjusted β: -0.72 [95% CI: -1.39, -0.05], *p* = 0.04). Among women, no significant difference was observed in the DVS between recipients of public assistance and non-recipients in either the crude or full-adjusted models (β: -0.49 [95% CI: -1.16, 0.19], *p* = 0.16; adjusted β: -0.19 [95% CI: -1.06, 0.68], *p* = 0.66). Among men, an interaction was observed between receiving public assistance and eating together (adjusted β: 1.41 [95% CI: -0.12, 2.94], *p* = 0.07). Although this interaction effect was not significant for women, the point estimate suggested a positive association (adjusted β: 0.73 [95% CI: -0.55, 2.01], *p* = 0.26). For the sensitivity analysis, we conducted propensity score matching and analyzed the relationship between DVS and receiving public assistance. A total of 66 matched pairs were established for men and 57 matched pairs for women. The GLM results of the sensitivity analysis showed that the association between receiving public assistance and food diversity remained largely unchanged, even among populations with the same standard of living, indicating consistency with the main analysis (Additional file 1). In the matched samples, although these associations did not reach significance, point estimates of the interaction term between public assistance and eating together showed a positive association among both men and women.

**Table 2:** Association between receiving public assistance, dietary variety score, and eating together

	Crude model			Full-adjusted model		
	β	95% CI	*p*	β	95% CI	*p*
Men						
Public assistance (ref. non-recipients)						
Recipients	-1.61	(-2.23, -0.98)	<0.001	-0.72	(-1.39, -0.05)	0.04
Eating together (ref. not everyday)						
Everyday				0.72	(0.57, 0.86)	<0.001
Recipients*Everyday				1.41	(-0.12, 2.94)	0.07
Women						
Public assistance (ref. non-recipients)						
Recipients	-0.49	(-1.16, 0.19)	0.16	-0.19	(-1.06, 0.68)	0.66
Eating together (ref. not everyday)						
Everyday				0.62	(0.45, 0.78)	<0.001
Recipients*Everyday				0.73	(-0.55, 2.01)	0.26

Abbreviations: CI, 95% confidence intervals; ref., reference; IADL, Instrumental Activities of Daily Living

We adjusted for age, living arrangement, marital status, education, household income, current medical treatment for diseases (hypertension, diabetes mellitus, hyperlipidemia, cancer, and depression), oral frailty risk, IADL, alcohol intake, smoking status, number of remaining teeth, and use of denture or bridge in the full-adjusted general linear model

## Discussion

In the present study, men receiving public assistance had lower food diversity than non-recipients, even after adjusting for sociodemographic factors. For women, no association was observed between receiving public assistance and a lower DVS. Food diversity was higher among men receiving public assistance and eating together daily.

Receiving public assistance was independently associated with a lower DVS, even when comparing individuals with the same standard of living, which may be explained by disparities in assets and other factors. We performed a sub-analysis after matching the background characteristics considered in applications for public assistance in Japan: household income, number of household members, and disease status (hypertension, diabetes mellitus, hyperlipidemia, cancer, and depression) between recipients of public assistance and non-recipients. After adjusting for these factors, a consistent trend was observed towards a lower DVS among man recipients. This may be because non-recipients with an income or medical conditions eligible for public assistance have assets that recipients do not have, the ability to manage daily life, or other factors that would not have led them to receive the assistance. Indeed, among older people, having more assets has been reported to be associated with higher food diversity [15].

Gender differences in the association between public assistance and DVS may be explained by differences in cooking skills. In a previous study, those who prepared meals from ingredients ate more balanced meals and breakfasts than those who relied on commercial foods or rarely cooked meals, regardless of their economic situation [40]. A study based on a national survey in Japan revealed that 20.1% of men and 79.9% of women prepared most of their daily meals from ingredients [40]. Women generally have better cooking skills than men, and limited cooking skills are associated with inadequate vegetable and fruit consumption [41]. Furthermore, the frequency of eating out was associated with a lower vegetable intake [42]. Indeed, single men with good cooking skills were more inclined to adhere to a healthy diet [43].

The independent association between eating together and a higher DVS among men may be explained by differences in the social support received. Previous studies have shown that older adults who routinely eat alone have a lower food diversity [25]. In contrast, the amount of instrumental support received, such as in preparing and receiving meals, has been associated with higher food diversity among older adults who live alone [16]. Therefore, instrumental support, such as the serving of meals, may be effective in improving the food diversity of recipients of public assistance.

The finding that receiving public assistance is associated with a low food diversity may have important policy implications. The results of this study indicate that financial assistance through the existing public assistance system has not been associated with improved food diversity. This suggests the need for additional support to improve the diets of recipients. The healthcare support program for recipients of public assistance has been mandated for welfare offices in municipalities in Japan since January 2021 [44]. As part of this support, providing healthy meals to recipients of public assistance and creating an environment that promotes healthy food intake, such as opportunities for communal meals in which recipients can participate, should be considered. For example, since public assistance recipients are prone to isolation [45], the communal meal place should be designed to remove psychological barriers and make it easier for the recipients to participate.

The present study is the first to demonstrate the dietary status of recipients of public assistance in an older population in Japan; however, it has some limitations. First, the study was conducted using self-administered questionnaires mailed to participants, and those with missing data were excluded, which may have introduced selection bias. While most variables used in the analysis had relatively low proportions of missing data (ranging from 0 to 7.9%), household income had a higher missingness rate of 13.9% (9.4% in men and 17.9% in women). The results may have been underestimated if more economically deprived participants were unable to respond. Second, the present study used a self-administered questionnaire, which may have led to differential misclassification. Owing to the stigma associated with receiving public assistance in Japan [46], the outcomes of the present study may have been underestimated if participants who received public assistance reported themselves as non-recipients. Additionally, recipients of public assistance are prone to social challenges, which could make it difficult for them to report food diversity accurately.

## Conclusions

In conclusion, even after adjusting for sociodemographic factors, men receiving public assistance demonstrated lower food diversity than non-recipients. Men receiving public assistance were more likely to increase their food diversity by eating together. To ensure recipient’s rights to food security, public assistance programs need to consider additional support beyond financial assistance, such as enabling recipients to eat together.

## Electronic supplementary material

Below is the link to the electronic supplementary material.

Supplementary Material 1: Association among receiving public assistance, dietary variety scores, and eating together after propensity score matching

## Data Availability

The data that support the findings of this study are available from the JAGES project but restrictions apply to the availability of these data, which were used under license for the current study, and so are not publicly available. Data are however available from the authors upon reasonable request and with permission of the JAGES project.

## Abbreviations

JAGES: Japan Gerontological Evaluation Study
IADL: Instrumental Activities of Daily Living
GLM: General linear model
CI: Confidence intervals
DVS: Dietary Variety Score
