# Supplementary material for: Public assistance program and food diversity among older people: a cross-sectional study using the Japan Gerontological Evaluation Study data: Public assistance program and food diversity
Source: Int J Equity Health. 2025 May 12;24:134. doi: 10.1186/s12939-025-02494-3 (PMC12067910; doi:10.1186/s12939-025-02494-3)
Supplement: Supplementary file 1 — Supplementary Material 1: Association among receiving public assistance, dietary variety scores, and eating together after propensity score matching [file 12939_2025_2494_MOESM1_ESM.docx]

**Additional table 1** Association among receiving public assistance, dietary variety scores, and eating together after propensity score matching

|  |  | Crude model | | | | | | |  | Full-adjusted model | | | | | | |
| --- | --- | --- | --- | --- | --- | --- | --- | --- | --- | --- | --- | --- | --- | --- | --- | --- |
|  |  | β |  | 95% CI | | |  | p |  | β |  | 95% CI | | |  | p |
| Men |  |  |  |  |  |  |  |  |  |  |  |  |  |  |  |  |
| Public assistance（ref. non-recipients） | | | | |  |  |  |  |  |  |  |  |  |  |  |  |
| Recipients |  | -1.12 | ( | -2.03 | , | -0.21 | ) | 0.02 |  | -1.63 | ( | -2.55 | , | -0.71 | ) | <0.001 |
| Eating together（ref. not everyday) | | |  |  |  |  |  |  |  |  |  |  |  |  |  |  |
| Everyday |  |  |  |  |  |  |  |  |  | 0.91 | ( | -0.50 | , | 2.32 | ) | 0.21 |
| Recipients*Everyday |  |  |  |  |  |  |  |  |  | 2.66 | ( | 0.72 | , | 4.60 | ) | 0.01 |
| Women |  |  |  |  |  |  |  |  |  |  |  |  |  |  |  |  |
| Public assistance（ref. non-recipients） | | | | |  |  |  |  |  |  |  |  |  |  |  |  |
| Recipients |  | -0.25 | ( | -1.17 | , | 0.68 | ) | 0.60 |  | -0.65 | ( | -1.88 | , | 0.58 | ) | 0.30 |
| Eating together（ref. not everyday) | | |  |  |  |  |  |  |  |  |  |  |  |  |  |  |
| Everyday |  |  |  |  |  |  |  |  |  | -0.52 | ( | -1.99 | , | 0.95 | ) | 0.30 |
| Recipients*Everyday |  |  |  |  |  |  |  |  |  | 0.88 | ( | -0.92 | , | 2.68 | ) | 0.34 |

We adjusted for household income, number of household members, and current medical treatment for diseases (hypertension, diabetes mellitus, hyperlipidemia, cancer, and depression) in the full-adjusted model.

CI, 95% confidence intervals; ref., reference.
